# Evidence in support of the role of disturbance vegetation for women’s health and childcare in Western Africa

**DOI:** 10.1186/1746-4269-10-42

**Published:** 2014-05-08

**Authors:** Alexandra M Towns, Sofie Ruysschaert, Esther van Vliet, Tinde van Andel

**Affiliations:** 1 Naturalis Biodiversity Center, Leiden University, Darwinweg 4, P.O. Box 9517, 2300, RA Leiden, The Netherlands; 2 Lab. Tropical and Subtropical Agriculture and Ethnobotany, Ghent University, Coupure Links 653, B-9000, Gent, Belgium; 3 Institute of Enviromental Biology, Utrecht University, Padualaan 8, 3584, CH Utrecht, The Netherlands

**Keywords:** Bénin, Gabon, Conservation, Disturbance vegetation, Medicinal plants, Non timber forest products, Sustainable extraction, Emic practices, Resilience, Socio-ecological systems

## Abstract

**Background:**

In savannah-dominated Bénin, West Africa, and forest-dominated Gabon, Central Africa, plants are a major source of healthcare for women and children. Due to this high demand and the reliance on wild populations as sources for medicinal plants, overharvesting of African medicinal plants is a common concern. Few studies in Western Africa, however, have assessed variations in harvest patterns across different ecological zones and within local communities.

**Methods:**

We investigated which vegetation types women accessed to harvest medicinal plants by conducting 163 questionnaires with market vendors and women from urban and rural communities. We made botanical vouchers of cited species and collected information on their vegetation type and cultivation status.

**Results:**

Secondary vegetation was a crucial asset; over 80% of the 335 Beninese and 272 Gabonese plant species came from disturbance vegetation and home gardens. In Bénin, access to trade channels allowed female market vendors to use more vulnerable species than rural and urban women who harvested for personal use. In Gabon, no relationship was found between vulnerable plant use and informant type.

**Conclusions:**

This study highlights the underemphasized point that secondary vegetation is an asset for women and children’s health in both savanna-dominated and forest-dominated landscapes. The use of disturbance vegetation demonstrates women’s resilience in meeting healthcare needs in the limited amount of space that is available to them. Species of conservation concern included forest species and savanna trees sold at markets in Bénin, especially *Xylopia aethiopica*, *Khaya senegalensis*, and *Monodora myristica*, and the timber trees with medicinal values in Gabon, such as *Baillonella toxisperma*.

## Background

Traditional medicine is the primary source of healthcare in Sub-Saharan Africa [1]. Herbal medicine in particular has a substantial role in sustaining the health of populations in both rural [2,3] and urban [4,5] communities in Africa. The trade of medicinal plants contributes to the informal economy in many African countries; estimates of the annual values range from US$ 64,000 in Sierra Leone to US$ 7.8 million in Ghana [6,7]. The profitability of the trade, combined with the frequent usage of medicinal plants and the reliance on wild populations [8,9], has generated concern from conservationists that frequently utilized species are being harvested at an unsustainable rate [10,11]. Estimations of the number of globally threatened medicinal plants range from 4,160 to 10,000 [12]. This concern is even more critical in areas of high conservation priority, such as the biologically diverse Congolian coastal forests of West and Central Africa [13], and is reflected in recent studies exploring the over-exploitation of medicinal plants in Bénin [14] and Cameroon [15].

However, little information is known on the ecology of many African medicinal plants [16]. If plants are harvested from primary forest vegetation or have a rare or endemic status, over-harvesting can be a particularly serious threat [17]. The collection of plants from disturbed vegetation, however, would have far less impact on the environment, since species in human-altered landscapes are generally fast-growing, have short life spans, and have a wide distribution [18]. Although plant harvesting may kill plant individuals, the great majority of disturbance species is abundant and has the ability to regenerate easily [19]. Although medicinal plant cultivation may not be entirely sustainable on the ecological level [8], cultivated species have a low risk of extinction due to their management by people.

Identifying differences in harvesting patterns is also important to consider when assessing the environmental impacts of herbal medicine extraction [12]. There is little research, however, from West and Central Africa that assesses variations in plant use patterns within one country [20] or across ecological conditions [14]. Plant use patterns can vary between different members of a community [21], especially between men and women [22]. Studies in Madagascar and Ivory Coast found that women had more knowledge of plants from village surroundings and buffer zones than from the forest [23,24]. There is also a distinction between plant harvesting for commercial and subsistence use; the commercialization of medicinal plants has been documented as a greater threat to plant biodiversity [12], especially given the demand by growing African urban populations [10]. Pinpointing which vegetation types are utilized by different members of a community is an important foundation for identifying conservation priorities, designing environmental management programs, and understanding how local populations manage their health. There may be considerable variation in the vegetation types that are utilized by different community members, with substantially different impacts on the environment.

In order to bridge the gaps in understanding African medicinal plant ecology and women’s plant use patterns, we worked in two ecologically-diverse countries in Western Africa: savanna-dominated Bénin and forest-dominated Gabon. We focused on the following research questions: *Which vegetation types are major sources of herbal medicine for women and children in Bénin and Gabon? What are the differences in plant use patterns between herbal medicine vendors and urban and rural women who harvest for personal use?* We defined the domain of women’s knowledge as plants that are used for women’s health and childcare. We expected all women to harvest predominantly from secondary forest and disturbance vegetation on the basis of women’s specific knowledge of plants from human-altered vegetation in other parts of Africa [23,24]. We expected rural women to use more vulnerable and primary forest species than urban and market women due to the proximity of rural communities to primary forest vegetation.

## Methodology

### Research sites

Bénin is located in the West African Dahomey gap, a savannah corridor between the Lower and Upper Guinea forests. The Beninese landscape is 50% savannah and 2.5% gallery forest [25]. It has recorded levels of high deforestation [26], with the remaining mosaic forest clusters and forested savannah scattered across the south of the country, housing 20% of the country’s flora and 64% of its threatened species [27]. From April to October 2011, we carried out research in the eight southern-most departments of Bénin: Collines, Zou, Plateau, Kouffo, Mono, Atlantique, Littoral, and Oueme. We chose these departments on the basis of the high concentration of people, especially the ethnic majorities Fon and Yoruba, and the large number of medicinal plant markets in this region [28]. We worked with mainly Fon and Yoruba ethnic groups in rural, urban, and marketplace settings within these eight departments.

Gabon borders the Atlantic Ocean at the Equator, between Republic of the Congo and Equatorial Guinea. It is estimated that over 80% of Gabon is covered with forest [29], with up to 65% of the forest considered primary [30]. It currently has the highest loss of primary forest in Africa [30]. The remaining land area is comprised of swamps, mangroves, and savannas [31]. Research in Gabon was completed between June and December 2012, spanning the six provinces of Estuaire, Wolem-Ntem, Haut-Ogooué, Ngounié, Moyen Ogooué, and Ogooué-Ivindo. We worked in rural, urban and market settings with Bantu-speaking ethnic groups.

### Data collection

The research team worked within the Code of Ethics of the International Society of Ethnobiology [32], followed all protocols with partner institutions, and obtained formal invitations, research permits, and plant export permits. We carefully explained the nature of our research and obtained prior informed consent from all participants. We initiated our data collection at the marketplace, speaking informally with herbal medicine saleswomen and purchasing plants in order to familiarize ourselves with local healthcare priorities and commonly utilized medicinal plant species. We then utilized snow-ball sampling to identify additional women from the markets and women from urban and rural communities with whom we conducted our ethnobotanical questionnaires. Based on standard ethnobotanical methods [33], the questionnaires included free-listing exercises on common maternal and infant health ailments and structured questions on herbal recipes to treat specific illnesses.

In Bénin, we conducted a total of 85 ethnobotanical questionnaires, 42 on women’s health and 43 on childcare. The 85 questionnaires were carried out with 48 market vendors, 27 women from rural communities, and 10 women from urban communities. We worked with the following ethnic groups: Fon and related (66%), Yoruba and related (15%), Adja and related (6%) and mixed ethnicities (13%). In Gabon, we conducted a total of 78 ethnobotanical questionnaires, 40 on women’s health and 38 on childcare, distributed as follows: 56 with women from rural communities, 12 market vendors, and 10 women from urban communities. We worked with the following ethnic groups: Fang (43%), Mitsogo (15%), Babungu (15%), Obamba (8%), Ossimba (4%), Bapounou (4%), and other (11%). We defined urban settings as those communities with a population larger than 35,000 people, including the Beninese cities of Abomey, Abomey-Calavi, Cotonou, Dassa, Lokossa, Pobe, and Porto-Novo and the Gabonese cities of Libreville, Franceville, and Oyem. Rural communities in which we worked included the villages surrounding these areas, with populations no larger than 6000 people. Interpreters were hired to translate local languages into French. The questionnaires in market settings took place in market stalls during regular business hours. In rural and urban locations, the questionnaires took place in the homes or businesses of the informant. All informants were given monetary compensation equivalent to local norms for their participation in the research.

Immediately after the completion of each questionnaire, informants led the research team on plant collection walks, resulting in the collection of over 1500 botanical specimens. We collected plant specimens for all cited local plant names following standard botanical methods. After successfully pairing the local name of a plant to a corresponding collection for later identification, we only made additional collections of repeated species when in doubt [34]. We purchased plants cited by saleswomen directly on the market, and later accompanied the women into the field to match market specimens with their corresponding species in the wild. Duplicates of collected specimens were deposited at the Herbier National du Bénin (BEN) and the Herbier National du Gabon (LBV). A full set of specimens was deposited at the Wageningen branch of the National Herbarium of the Netherlands, now part of Naturalis Biodiversity Center (L).

### Data analysis

We entered data acquired through the questionnaires into a database, which included scientific names, local names, plant part, preparation, recipe, and informant type. Identical local names within the same language were matched with the same corresponding scientific name of identified collections. We matched 98% of the Beninese database and 93% of the Gabonese database with scientific nomenclature. The remaining unidentified plants from each country were excluded from further analyses. The research data were then classified into vegetation type by means of our own observations in the field and botanical literature [35-39]. We divided the plants into five vegetation types: primary forest, disturbance vegetation- including secondary forest and shrubland around villages, savanna, mangroves/wetlands, and cultivated- including both wild plants taken from their natural surroundings and planted in home gardens and true domesticated species such as *Zea mays*. We also recorded the conservation status of each species on the International Union for Conservation of Nature (IUCN) Red List [40], Red List for Bénin [27] and the Convention on International Trade of Endangered Species (CITES) list [41].

We conducted cluster analyses for each country to assess the similarity of informants’ responses. All plant species cited by informants were entered into presence–absence data matrices for each country. We performed a Detrended Correspondence Analysis (DCA) in PC-ORD v 5.33, which identified the two main axes that caused the distribution of our informants and cited species [42]. We plotted the 1st and 2nd axes in two-dimensional graphs to visualize the variation and overlap in plant species used by different informant types, making one comparison between women’s health and childcare informants and a second comparison between rural, urban, and market informants. Using Statistica version 8.0, we performed Kruskall-Wallis tests to assess whether women used plants mainly from secondary vegetation, and whether rural women used more vulnerable species than urban or market women. Vulnerable species were defined as primary forest species and those species that were included on the Bénin Red List, CITES, and/or IUCN Red list.

## Results

### Most commonly cited species and vegetation types

In Bénin we recorded a total of 335 medicinal plant species from 87 families used for women and children. The plants were found in the following vegetation types: 57% disturbance vegetation, 30% cultivated, 19% savannah, 7% primary forest, and 6% wetlands and mangroves. The percentages totaled higher than 100% because some plants occurred in multiple vegetation types. Forty-nine (26%) of the 188 species originating from secondary vegetation were considered weeds in literature [35,38,43]. Combining wild species found in disturbance vegetation and cultivated plants, 87% of species used for women’s health and childcare were harvested from human-disturbed habitats. At most 32% of mentioned species were found also in undisturbed vegetation (savanna, primary forest and wetlands). However, those plants that occurred both in primary forest and disturbance vegetation, such as *Baphia nitida*, were most likely not harvested from the forest (too far away), thus only 12 species (4%) were exclusively harvested from undisturbed forest because they did not occur elsewhere. The most commonly cited Beninese species were largely cultivated or harvested from secondary vegetation (Table 1).

**Table 1:** Most frequently cited plant species in Bénin from 43 childcare (CC) questionnaires and 42 women’s health (WH) questionnaires

**Species**	**Vegetation type**	**Conservation status**	**CC frequency [%]**^**c**^	**WH frequency [%]**^**c**^
*Ocimum gratissimum* L.	disturbance, cultivated		72	62
*Sarcocephalus latifolius* (Sm.) E.A. Bruce	savanna		33	69
*Securidaca longipedunculata* Fresen.	savanna		44	57
*Momordica charantia* L.	disturbance		60	21
*Citrus aurantifolia* (Christm.) Swingle	cultivated		44	36
*Xylopia aethiopica* (Dunal) A.Rich.	disturbance	VU^b^	56	19
*Ocimum* sp.	cultivated		26	40
*Khaya senegalensis* (Desv.) A.Juss.	savanna, cultivated	VU^a^, EN^b^	30	36
*Dichapetalum madagascariense* Poir.	disturbance		28	29
*Schwenckia americana* L.	disturbance		26	29
*Uvaria chamae* P.Beauv.	disturbance		16	36
*Heterotis rotundifolia* (Sm.) Jacq.-Fél.	disturbance		28	24
*Senna siamea* (Lam.) H.S.Irwin & Barneby	disturbance, cultivated		35	17
*Monodora myristica* (Gaertn.) Dunal	disturbance	EN^b^	35	17
*Caesalpinia bonduc* (L.) Roxb.	disturbance, cultivated	EW^b^	21	29
*Allium sativum* L.	cultivated		30	19
*Carica papaya* L.	cultivated		26	21
*Argemone mexicana* L*.*	disturbance		35	12
*Psidium guajava* L.	cultivated		44	2

^a^IUCN.

^b^Bénin Red List [27].

^c^The number of informants who mentioned each species at least once per questionnaire divided by the total number of informants.

*VU* = vulnerable; *EN* = endangered; *EW* = extinct in wild.

In Gabon we recorded a total of 272 medicinal plant species from 84 families used for women’s and children’s health. Of these species, 79% came from secondary forest or disturbed vegetation, 20% from cultivated sources, 17% from primary forest, 3% from savannah, and 2% from wetlands and mangroves. Thirty-four (16%) of the 215 secondary vegetation species were considered weeds in literature [35,38,43]. At most 22% of the species were found in undisturbed vegetation, with 13 species (5%) exclusively harvested from primary forest. While many of the most commonly cited Gabonese species originated from secondary vegetation or cultivation (Table 2), more commonly cited Gabonese species came from primary vegetation than Beninese species.

**Table 2:** Most frequently cited plant species in Gabon from 38 childcare (CC) questionnaires and 40 women’s health (WH) questionnaires

**Species**	**Vegetation type**	**IUCN status**	CC frequency [%]^*^	WH frequency [%]^*^
*Citrus aurantifolia* (Christm.) Swingle	cultivated		74	10
*Capsicum annuum* L.	cultivated		39	30
*Manihot esculenta* Crantz	cultivated		45	23
*Pterocarpus soyauxii* Taub.	primary		34	28
*Elaeis guineensis* Jacq.	disturbance, cultivated		37	23
*Musa* sp.	cultivated		37	20
*Annickia affinis* (Exell) Versteegh & Sosef	primary, disturbance		37	18
*Costus* sp.	disturbance		37	18
*Alchornea cordifolia* (Schumach. & Thonn.) Müll.Arg.	disturbance		8	40
*Harungana madagascariensis* Lam. ex Poir.	disturbance		34	8
*Psidium guajava* L.	cultivated		37	5
*Aframomum* sp.	disturbance		29	10
*Cola* sp.	disturbance		32	8
*Cucumeropsis mannii* Naudin	disturbance, cultivated		18	20
*Pentaclethra macrophylla* Benth.	primary, disturbance		18	20
*Ocimum gratissimum* L.	disturbance, cultivated		13	18
*Baillonella toxisperma* Pierre	primary	VU	13	18
*Alstonia* cf. *boonei* De Wild.	primary, disturbance		29	0
*Sida acuta* Burm.f.	disturbance		3	25
*Vernonia amygdalina* Delile	disturbance, cultivated		24	3
*Aframomum melegueta* K.Schum	disturbance, cultivated		21	5
*Mangifera indica* L.	cultivated		16	10
*Senna alata* (L.) Roxb.	disturbance		16	10
*Alstonia congensis* Engl.	wetlands		0	25

*Number of informants who mentioned each species at least once per questionnaire, divided by the total number of informants.

*VU* = vulnerable.

### Species with priority conservation status

Beninese species which were commonly cited and also figured on conservation lists as species of concern included: *Xylopia aethiopica*, *Khaya senegalensis*, *Monodora myristica*, and *Caesalpinia bonduc*. We observed, however, that *C. bonduc* seeds and leaves and *K. senegalensis* bark were often harvested from cultivated sources. *X. aethiopica* and *M. myristica* were cited almost exclusively by market informants. The only CITES species mentioned in our study, *Aloe marcocarpa*, was mentioned one time by a market informant. In Gabon, one of the most frequently cited species, the highly valued timber tree *Baillonella toxisperma*, is considered vulnerable by the IUCN (Table 2). No Gabonese species from our study figured on the CITES list.

### Differences in plant use among rural, urban and market women

**Figure 1:**
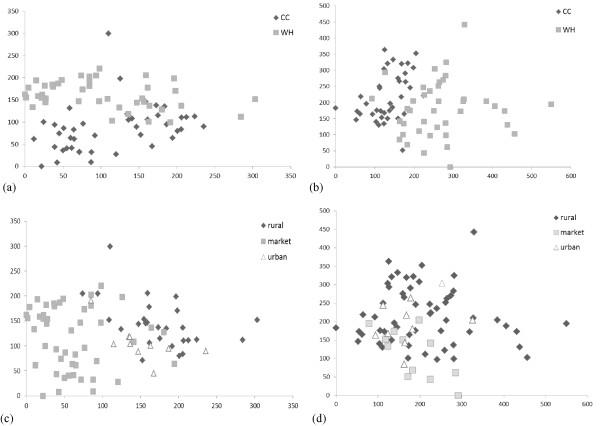
DCA scatterplot comparing women’s health informants and childcare informants on species level for (a) Bénin (N = 85) and (b) Gabon (N = 78) and plant use patterns among market, urban, and rural informants for (c) Bénin and (d) Gabon.

In Bénin, there was a slight overlap in species cited for women’s health and childcare (Figure 1a), with a somewhat larger variation in plants cited for childcare than for women’s health. In Gabon, species between the two health categories also overlapped (Figure 1b), but a larger variation in plants was cited for women’s health than for childcare. When both health categories were combined and plants used among different informant groups were compared, rural and urban women from Bénin largely cited the same plants, while market women cited a wider variety of species with less agreement (Figure 1c). Urban Gabonese women cited a subset of the wide variety of species cited by rural women (Figure 1d). Market women in Gabon cited largely the same species as rural women, with some variation. Our results suggest that market women in Bénin and rural women in Gabon use the greatest diversity of plant species.

All women, regardless of informant type and country, used more species from disturbance vegetation than from any other vegetation type (p < 0.001). Beninese market women used more vulnerable species than rural or urban women (p = 0.0288). Rural and urban women used about the same amount of vulnerable species. When we further divided the market informants into vendors from the large metropolitan markets of Cotonou and Porto Novo (n = 22), and merchants from all other regional markets (n = 26), we found that metropolitan market women used more primary forest species than all other informants (p = 0.0016), while regional market vendors’ plant citations resembled rural and urban informants. Rural Gabonese women did not use significantly different numbers of vulnerable plant species than urban or market women (p = 0.7450).

## Discussion

### Space matters: the spatial dimension of medical plant harvesting

Our research highlights the importance of secondary vegetation in two ecologically diverse countries. Bénin is covered for more than 50% by savannah, yet only 19% of medicinal plants for women and children come from savannah vegetation. Gabon is covered with 65% primary forest, yet only 17% of women’s and children’s pharmacopeias is harvested from primary forest. While it is not surprising that Gabonese women were more likely to incorporate primary forest species in their pharmacopeia than women from Bénin given the high percentage of primary vegetation available to them, both groups of women cited secondary vegetation most frequently. Although savannah and forest species were available, women in both countries predominantly used plants that were most closely surrounding them - species from disturbance vegetation and home gardens. Research from other tropical regions of the world, although not always specific to women’s plant use, emphasizes the role of disturbance vegetation in traditional pharmacopeias [44-49].

Our results support both the plant apparency theory [50] and the resource availability theory [51], originally used in herbivory studies to explain herbivores’ use of different species within an environment. These theories have been applied to human plant use patterns, to explain the selection by people of those plants that are most available to them [52-54]. While these theories are widely applied to explain the use of individual species or Non-Timber Forest Products (NTFPs) [55], our results support their application to a broader vegetation community [56], suggesting the most apparent and accessible plant communities are most likely to be used by human populations. The use of disturbance vegetation may also be explained by the chemical components needed by plants in disturbance areas to survive. The higher quantity of chemical properties in herbs and plants with short-life cycles are not needed by forest trees, which have evolved structural defense systems [46-49].

### Gender matters: women’s medicinal plant harvesting

The frequent citation of secondary vegetation and home gardens as sources of medicinal plants may be influenced by differences in gender. Men and women have different social and work-based roles [57] with distinct domains of knowledge. Men frequently work outside the village, while women’s activities tend to revolve in and around the home. These dissimilar activities result in the occupation of different spaces, and thus, different access to natural resources and vegetation types [58]. Given women’s high domestic workload, they likely do not have the time or means to travel to the rainforest to harvest medicinal plants. Plants that take several days to find and collect are not very beneficial to treat oneself nor one's sick children. These circumstances should be considered when designing and managing conservation programs. Women’s perspectives and the vegetation types that are most useful to them need to be included in the decision-making processes in order to ensure the equal distribution of benefits of forest management initiatives [21,22].

Although definitive claims on the sustainability of plant extraction would need to include population assessments, impact studies and measurements on the rate of extraction versus the rate of natural regeneration [9,16], generally speaking, when cultivated plants and species from human-altered vegetation types dominate a pharmacopeia and primary forest species are hardly used, plant extraction can generally be considered a non-destructive use of resources [19]. This does not mean, however, that all medicinal plant extraction in these countries follows a similar pattern. While women tend to use species associated with habitats of high human influence, men’s use of plants extends to all parts of the forest, including old-growth forested habitats [49,57]. There are indications that plants commonly used and known by men, such as aphrodisiacs or ritual plants, often come from primary forest [23,59]. More research is needed on male-dominated plant knowledge domains in order to assess men’s medicinal plant harvesting patterns and their impact on natural resources. In addition, variations of plant use patterns by women from different ethnic groups should be analyzed in future research.

### Commercialization matters: species for concern

The majority of species used and sold by market vendors in both countries came from human-altered habitats. Among all market vendors in Bénin, women who worked in large metropolitan markets, far removed from the primary forest, were the most likely to cite vulnerable species due to their access to the forest through trade. This access is made possible by the exploitation of wild plants of a larger area, including importing plants from other countries [60,61], and as a consequence, a higher variety of plant species including a higher chance of vulnerable species. In a forested country like Gabon, primary forest products were still widely available, including those who did not have access to trade networks. Including special consideration for the role of the market on the sustainability of medicinal plant extraction is essential to improved decision-making in land management and livelihoods [62].

Although the women in our study relied heavily on disturbance vegetation and cultivated species, we did find some exceptions in both countries. The frequently cited Beninese species *Xylopia aethiopica*, *Khaya senegalensis*, *Monodora myristica*, and *Caesalpinia bonduc* were identified as priority conservation species in market surveys in Bénin [28] and Ghana [7], although some of these products may come from cultivated sources. Although *C. bonduc* is considered extinct in the wild according to the Bénin Red List [27], we encountered it both cultivated in house yards and in disturbance areas, suggesting that some wild populations may still exist, although they may be intensively managed by local people. Recent research from Bénin has highlighted the overharvesting of *K. senegalensis*[63]. Although *Baillonella toxisperma* is the only frequently cited Gabonese species from our research on the IUCN red list*,* other primary forest species such as *Pterocarpus soyauxii* and *Annickia affinis* should also be further investigated for overharvesting issues [64]. Both *B. toxisperma* and *P. soyauxii* are valuable timber species exported from Central Africa [65].

### Linkage between local resource management and healthcare

Our results give insight into Beninese and Gabonese women’s emic practices of natural resource management and provision of healthcare. In the limited space that is available to a woman, she cares for her children and her own wellbeing by growing domesticated species, transplanting forest species in her home garden or cultivated field, and managing the vegetation in her immediate surroundings. The women have overcome the vulnerability of having access to a limited space and few resources to meet their healthcare needs, demonstrating the resilience and adaptation associated with socio-ecological systems in sustainable development literature [66,67]. If deforestation rates continue to increase, the loss of rainforest species may not severely impact the availability of medicinal plants for women and their children since they will likely continue to draw upon the resources easily accessible in disturbance and weedy vegetation, and will employ adaptive strategies to safeguard limited resources, such as we observed in the cultivation of vulnerable species like *Caesalpinia bonduc*.

## Conclusions

Our research emphasizes the role of disturbance vegetation in gynecological and pediatric healthcare in both savannah and forest-dominated landscapes in Western Africa. It demonstrates women’s resilience in meeting healthcare needs in the limited geographic space that is available to them and suggests their ability to adapt in the face of future deforestation. It also highlights substantial differences in plant use practices between women who sell plants commercially and women who harvest plants for personal use, particularly in the case of market women in Bénin. Although our research suggests that Western African women’s medicinal plant harvest can be considered generally sustainable due to their heavy reliance on human-altered habitats, additional research is needed on the ecology and regeneration of medicinal plant species in order to make specific conclusions on the sustainability of their harvest. Conservation efforts should mainly focus on Gabonese timber trees, especially *Baillonella toxisperma* and *Pterocarpus soyauxii* and the commonly sold market species in Bénin, *Xylopia aethiopica, Khaya senegalensis, Monodora myristica*, and *Caesalpinia bonduc*.

## Consent

Oral informed consent was obtained from all participants for the publication of this report.

## Abbreviations

BEN: Herbier National du Bénin; LBV: Herbier National du Gabon; L: Naturalis Biodiversity Center; IUCN: International union for conservation of nature; CITES: Convention on international trade of endangered species; DCA: Detrended correspondence analysis; NTFPs: Non-timber forest products.

## Competing interests

The authors state that they have no competing interests.

## Authors’ contributions

AMT carried out the ethnobotanical questionnaires in Bénin and Gabon, collected and identified the plants, analyzed the data, and drafted the manuscript. SR helped conduct the questionnaires and collect plants in Bénin and contributed to revising the manuscript. EvV helped to conduct the questionnaires, collect and identify plants in Gabon, and revised the manuscript. TvA conceived of the study, acquired funding, participated in its design and coordination, helped to identify the plants, and helped to draft the manuscript. All authors read and approved the final manuscript.
